# Study of the Process of Calcium Sulfide-Based Luminophore Formation from Phosphogypsum

**DOI:** 10.3390/molecules29225486

**Published:** 2024-11-20

**Authors:** Marina A. Egorova, Daniil I. Monastyrskiy, Oleg A. Medennikov, Nina P. Shabelskaya, Zlatislava D. Khliyan, Vera A. Ulyanova, Sergey I. Sulima, Elena V. Sulima

**Affiliations:** 1 Department of Ecology and Industrial Safety, Faculty of Technology, Platov South-Russian State Polytechnic University (NPI), Novocherkassk 346428, Russia; m.egorova@npi-tu.ru (M.A.E.); danya.monastyrskij.95@mail.ru (D.I.M.); monomors@yandex.ru (O.A.M.); nina_shabelskaya@mail.ru (N.P.S.); zlata.tkachenko.98@mail.ru (Z.D.K.); u.vera20@yandex.ru (V.A.U.); 2 Department of Chemical Technologies, Faculty of Technology, Platov South-Russian State Polytechnic University (NPI), Novocherkassk 346428, Russia; elena-sulima66@mail.ru

**Keywords:** alkaline earth metal sulfates, luminescence mechanism, crystal defects, ultraviolet pigments, phosphogypsum

## Abstract

One of the priority goals of sustainable socio-economic development for the period up to 2030 is providing food for the planet’s population. This entails an increase in the output of mineral fertilizers and, consequently, an increase in the quantities of solid industrial waste. Phosphogypsum, a by-product of phosphate fertilizer production from apatite ore, is one example of such waste. The problem of solid industrial waste recycling is urgent. The present study examines the process of converting calcium sulfate, in the form of a reagent, and phosphogypsum into a composite material of calcium sulfate/sulfide. An environmentally friendly material, sucrose, is used as a reducing agent. Reduced phosphogypsum (as well as calcium sulfate) luminescence is suggested to be associated with the formation of a CaS/CaSO_4_ composite material. The synthesized materials are characterized by X-ray phase analysis, X-ray photoelectron spectroscopy, elemental analysis, and calcium sulfide qualitative and quantitative content in the samples. It is shown that in the reduction process at the phase contact point, crystal grids are formed with a significant number of defects, which contributes to the convergence of some of the energy levels of the calcium cation and sulfide anion, facilitating the transitions of electrons from the valence zone to the core zone and the formation of luminescence centers (cross-luminescence). Both samples of reduced phosphogypsum and alkaline earth metal sulfates are found to exhibit luminescence properties under ultraviolet radiation. The data obtained open up broad prospects for the use of solid industrial waste for the synthesis of new materials.

## 1. Introduction

One of the priority goals of sustainable socio-economic development for the period up to 2030 is providing food for the planet’s population. This entails an increase in the production of mineral fertilizers and, consequently, an increase in the quantities of solid industrial waste. Phosphogypsum, a by-product of the production of phosphorus fertilizers from apatite ore, is one example of such waste. Numerous publications are devoted to the problem of solid industrial waste processing [1,2,3,4,5], which indicates the urgency of such research. At present, studies aimed at obtaining affordable new products from industrial waste are of particular importance.

The process of converting calcium sulfate into sulfide has been discussed many times in the scientific literature [6,7,8]. Earlier works [7,8] demonstrated the possibility of creating a luminescent material from phosphogypsum.

The phenomenon of cold glow or luminescence differs fundamentally from thermal radiation, since in this case, when additional energy is imparted to them, substances begin to radiate at lower temperatures when the available thermal energy cannot fully explain the processes that occur. An example of such a process is the glow of clock face numbers in the dark. The term “luminescence” was proposed by E. Wiedemann to describe such a glow, which occurs as a result of electron transitions in a material system.

It is known that inorganic luminescent materials such as silicates, phosphates, sulfides, and other compounds are often used in industry. In particular, sulfides of various metals such as zinc, barium, calcium, and cadmium are used to create luminescent glowing materials, which can be activated by various elements such as copper, silver, and rare earth metals. These materials are used in the manufacturing of various reflective and light-emitting devices such as traffic lights, luminous stickers, luminescent paints, and much more. Sulfides [9,10,11] are widely used as a luminophore. Doping the calcium sulfide structure with cations of various elements causes changes in the glow spectrum. For example, europium cations cause red [12,13,14,15] and orange [12] glow, and cerium can cause green and yellow-green glow [14]. Such doped materials find application in various light-emitting devices and products where it is important to control the color and brightness of the glow.

The process of sulfide-containing matrix synthesis can be carried out by both direct and indirect methods. In the case of the direct synthesis method, the thermolysis reaction is carried out between the process participants in a finely dispersed state in a hermetically sealed ampoule, usually filled with an inert gas. This method is usually used to create certain material structures with specified properties. Such high-temperature reactions allow for the obtaining of sulfide matrices with a certain composition and structure required for specific purposes and applications in various fields, including electronics, optics, and other technologies. The process of direct synthesis of sulfides, in which a hermetically sealed ampoule with reagents is placed in a one- or two-zone furnace, is distinguished as an important stage in the production of sulfide matrices. In this case, the gradual heating of the system plays a key role: the higher the temperature, the faster the reaction proceeds, and the homogeneity of the final compound increases. This approach helps to ensure a more stable and high-quality product. The reaction in Equation (1) depends on the specific reagents and synthesis conditions, and can be represented in the following general form for this process:

*a* Me + *b* S = Me_a_S_b_ (1)

Notably, the indirect method for obtaining alkaline earth metal sulfides differs from the direct method described above by a simpler process organization. In its case, sulfides are obtained from a number of compounds—salts and oxides—during their heterophase interaction with heated vapors of a specially introduced sulfonating agent. This method allows for the effective conversion of the initial compounds into sulfides without the high-temperature reactions, required by the direct method. This approach may be simpler and more cost-effective in certain situations, especially when synthesizing small product quantities or on a laboratory scale. In some cases, the hydrothermal method is used [9,15].

The process of alkaline earth metal sulfide formation by solid carbonate element exposure to a gaseous mixture of hydrogen sulfide and hydrogen at the reaction system temperature of 900 °C is industrially important for the synthesis of these compounds. Additional heating of the system by a pure hydrogen stream is often used to decompose possible polysulfide impurities. This process is lengthy; with a carbonate amount of 3–5 g, it usually takes about 2 h to complete the sulfidization process. This method ensures high-purity rare earth sulfides and is convenient for laboratory work or small-scale production. However, it is important to consider all technical aspects and conditions of the process to ensure efficiency and obtain the desired final product [16]. In [6], the process of processing galvanic sludge is presented with a description of the phenomenon of self-sulfurization.

The method of obtaining powdered calcium sulfide by heating CaCO_3_ with excess crystalline elemental sulfur S in a closed crucible at the firing temperature of 700 °C is also effective. This method, proposed in [17], allows for obtaining calcium sulfide using elemental sulfur as a reagent. The process is carried out in a closed crucible to ensure controlled reaction conditions. The temperature of 700 °C allows for activating the reaction between calcium carbonate and elemental sulfur, leading to the calcium sulfide formation. This method can be effective, especially in laboratory conditions, for obtaining calcium sulfide with desired properties. An unpleasant moment when using such a method is the presence of uncontrolled impurities of polysulfides.

In [18], the heat treatment method for a batch containing carbonates, sulfur, and a reducing agent, such as starch, is used to synthesize several alkaline earth element sulfides. The described approach allows for obtaining sulfides with high purity and improved properties. The main reaction that occurs during the synthesis of sulfides is described in [19] (Equation (2)):

4 MeCO_3_ + 4 S = 3 MeS + MeSO_4_ + 4 CO_2_↑ (2)

It is important to control side reactions, such as sulfate formation of the corresponding metal during sulfide synthesis, which can lead to undesirable products. To eliminate sulfates, they must be converted to sulfides. In this case, the introduction of a reducing agent that decomposes into carbon at high temperatures is a key strategy. This process is useful for removing sulfates and ensuring the desired product, the metal sulfide.

In [20], it was shown that crystalline calcium sulfide with a high degree of purity can be obtained from the crystal hydrate CaSO_4_∙2H_2_O. For this, 20 g of calcium sulfate in powder form was placed in a quartz boat, uniformly heated to 500–700 °C, and isothermally held for an hour in a hydrogen stream. This method ensures the production of finely crystalline calcium sulfide with a high degree of purity from the original sulfate powder under stable conditions.

The mechanism of light-emitting center formation is usually associated with doping atoms’ presence [11,15], which contributes to the change in the crystal grid energy structure and, consequently, to the luminescence formation in the material. However, the origin of light emission that occurs due to defects in the crystal grid is still unclear. Clarification may require further research aimed at studying the structure and properties of luminescent materials obtained from phosphogypsum in order to understand the exact nature of light emission in such materials and optimize their properties for specific applications.

The current study aims to investigate the luminescence process of the material obtained during the reductive heat treatment of calcium sulfate and phosphogypsum.

## 2. Results and Discussion

### 2.1. Sample Structures

**Figure 1:**
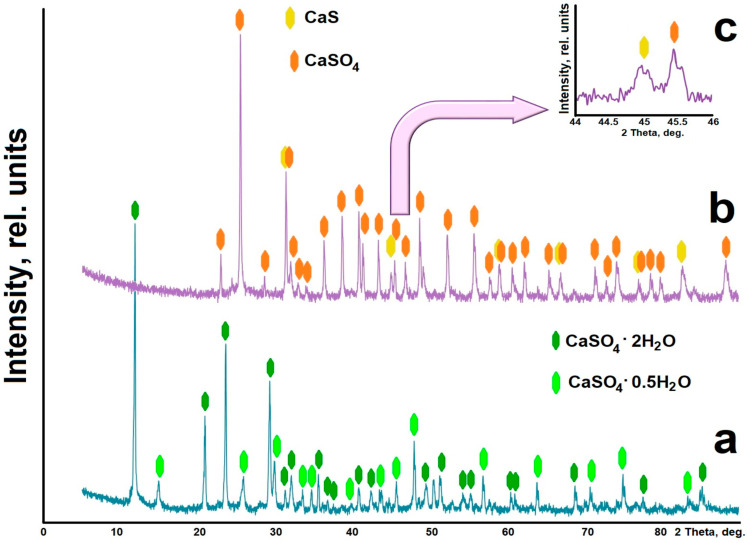
X-ray diffraction patterns of phosphogypsum samples: (**a**)—not heat-treated; (**b**) and (**c**)—heat-treated in the presence of a reducing agent; (**c**)—details.

The X-ray diffraction pattern of the reduced phosphogypsum (Figure 1b) shows that the sample contains peak characteristics of the calcium sulfate phase (CaSO_4_, PDF# 010-71-4906). In addition, reflections that can be attributed to the calcium sulfide phase (calcium sulfide, oldhamite, PDF# 010-72-0261) were found. Figure 1c shows the details of the X-ray diffraction pattern in the range of 44–46 2 Theta values. When analyzing the X-ray diffraction patterns, it is evident that during the heat treatment, the crystalline water is separated, and the dihydrate CaSO_4_·2H_2_O (calcium sulfate hydrate gypsum, calcium sulfate (VI) dihydrate, PDF# 010-70-7008) and hemihydrate CaSO_4_·0.5H_2_O (bassanite, calcium sulfate hydrate, PDF# 010-80-1235) gypsum (Figure 1a) are transformed into the anhydrous phase of calcium sulfate CaSO_4_. In this case, under the action of the reducing agent, the transition CaSO_4_ → CaS occurs. Part of the calcium sulfide, which is apparently weakly bound to the rest of the substance of the composite material, can be separated by chemical methods. This can be accomplished through appropriate chemical processes that separate the calcium sulfate and sulfide phases to obtain the desired components in the pure form. However, calcium sulfate or calcium sulfide alone does not exhibit luminescence at room temperature, only in a mixture in certain quantities.

### 2.2. Elemental Analysis

**Figure 2:**
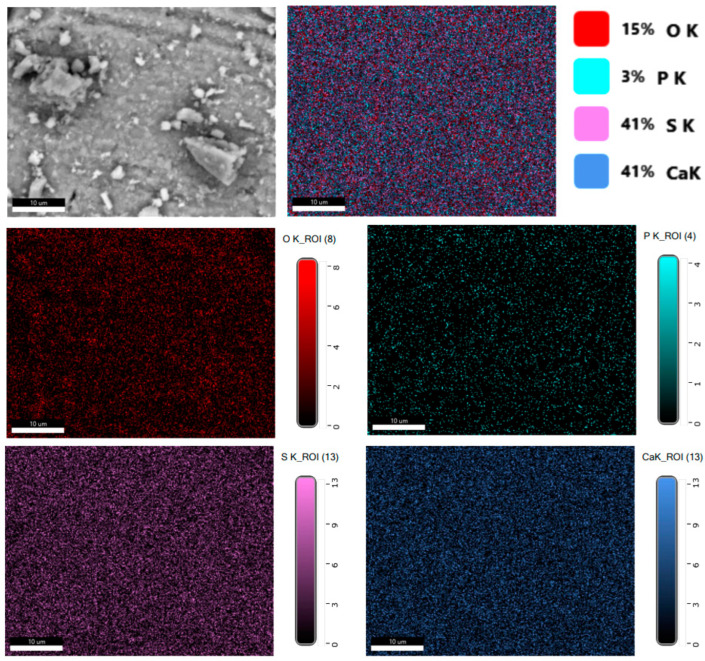
Distribution of elements in a reduced phosphogypsum sample.

Figure 2 shows the distribution of elements in a reduced phosphogypsum sample. The data analysis indicates a uniform distribution of elements in the sample; for calcium and sulfur, the field is more homogeneous, and for oxygen, there are areas where this element is less.

When material is formed during phosphogypsum heat treatment in the presence of a reducing agent, the oxygen content in the environment of the sulfur atom will consistently decrease with a simultaneous decrease in its oxidation state (reactions (3)–(6), formulas of substances that are not currently isolated in a free state are given in brackets):

CaS^+6^O_4_ + CO = CaS^+4^O_3_ + CO_2_ (3)

CaSO_3_ + CO = (CaS^+2^O_2_) + CO_2_ (4)

(CaSO_2_) + CO = (CaS^0^O) + CO_2_ (5)

(CaSO) + CO = CaS^−2^ + CO_2_ (6)

**Figure 3:**
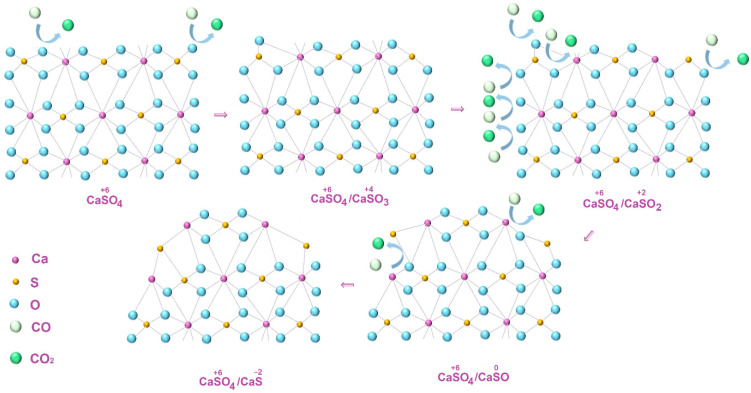
Schematic representation of calcium sulfate to sulfide partial reduction process.

The studied process of calcium sulfide sample formation can be schematically represented as follows (Figure 3).

**Figure 4:**
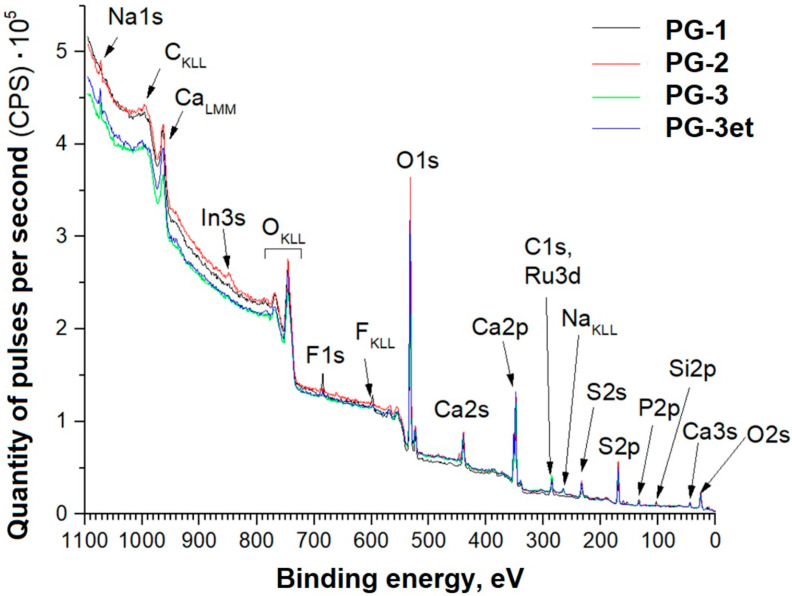
Panoramic spectra of phosphogypsum samples.

Panoramic XPS spectra of phosphogypsum samples with the detected chemical elements lines are indicated in Figure 4. For easier perception, the samples are designated as follows:

PG-1—original phosphogypsum.

PG-2—phosphogypsum subjected to heat treatment at a temperature of 900 °C (with a rate of temperature increase of 13 °C/min and isothermal holding for 60 min).

PG-3—phosphogypsum subjected to heat treatment in the presence of a reducing agent at a temperature of 900 °C (rate of temperature increase of 13 °C/min and isothermal holding for 60 min).

FG-3et—PG-3 sample, additionally subjected to argon ion etching with an energy of 1 keV (ion beam current of 10 mA) for 1 min.

The detected sodium and fluorine could have been introduced into the samples during sample preparation and were not considered when calculating the concentrations. The appearance of a weak indium line on one of the samples is due to indium use for preparing powder samples for analysis. Based on the data obtained, the concentrations of the detected elements in the surface layers of the provided samples were calculated (Table 1).

**Figure 5:**
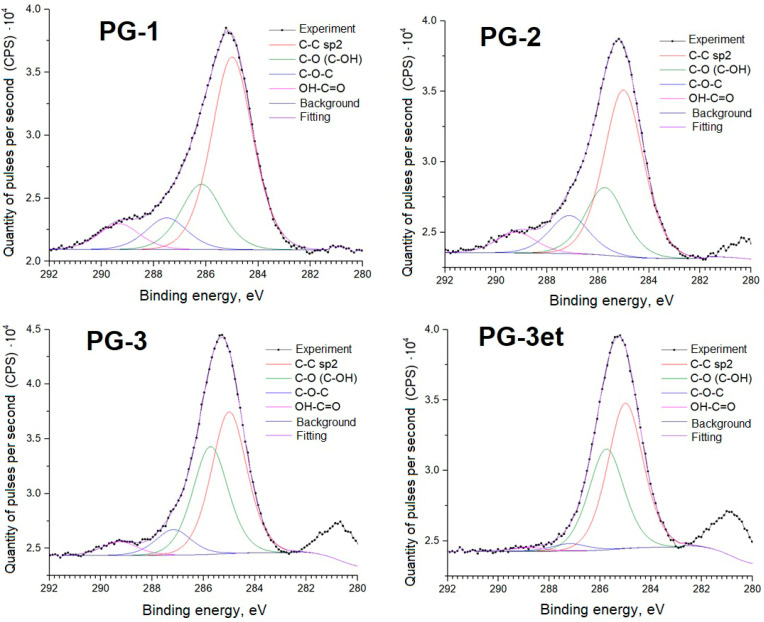
C1s spectra of phosphogypsum samples.

**Figure 6:**
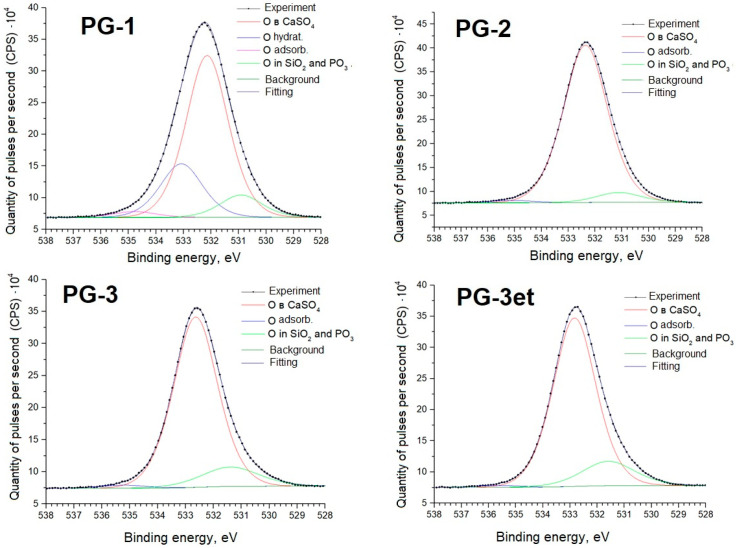
O1s spectra of phosphogypsum samples.

**Figure 7:**
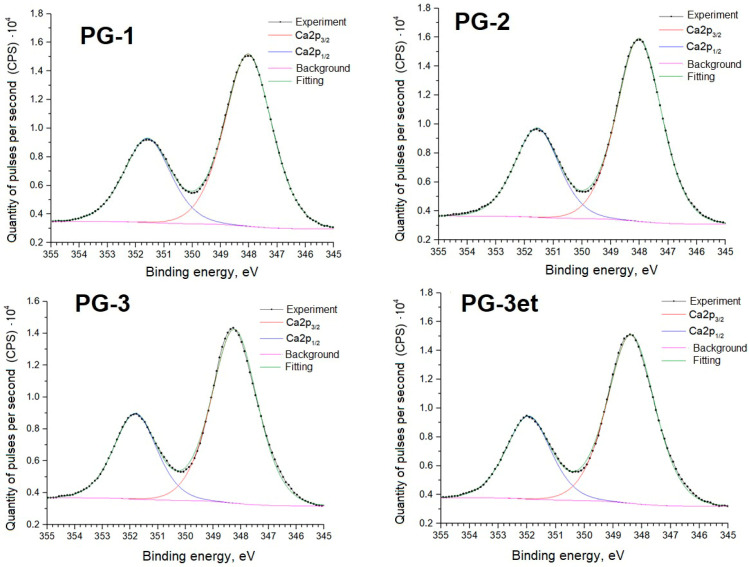
Ca2p spectra of phosphogypsum samples.

**Figure 8:**
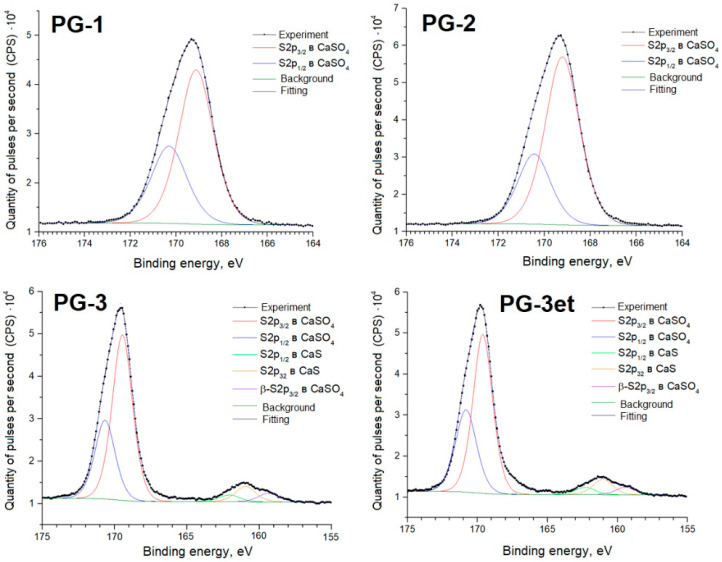
S2p spectra of phosphogypsum samples.

Further, detailed spectra of the samples and parameters of spectral decomposition into components with an indication of the corresponding chemical bonds are given (Figure 5, Figure 6, Figure 7 and Figure 8).

The C1s spectra of carbon adsorbed on the surface show a set of lines typical for powder samples. Samples PG-2 and PG-3 have a Ru3d line in the Eb region from 280 to 282 eV.

The O1s spectra show lines (Eb = 534–535 eV) associated with the presence of adsorbed water. The PG-1 sample spectrum shows a line (Eb = 533 eV) associated with water bound in the CaSO_4_·2H_2_O crystal hydrate.

The positions and intensity ratios of the Ca2p and S2p lines of the PG-1 and PG-2 samples fully correspond to the reference values for calcium sulfate CaSO_4_ [21]. In this compound, sulfur is in the oxidation state of +6. The PG-3 sample additionally exhibits S2p lines related to calcium sulfide (CaS) (sulfur with an oxidation state of −2) [22]. The proportion of sulfate relative to sulfide is 91.6/8.4 both before and after argon ion etching.

To clarify the luminescence center nature, similar studies were carried out for calcium sulfate (reagent).

**Figure 9:**
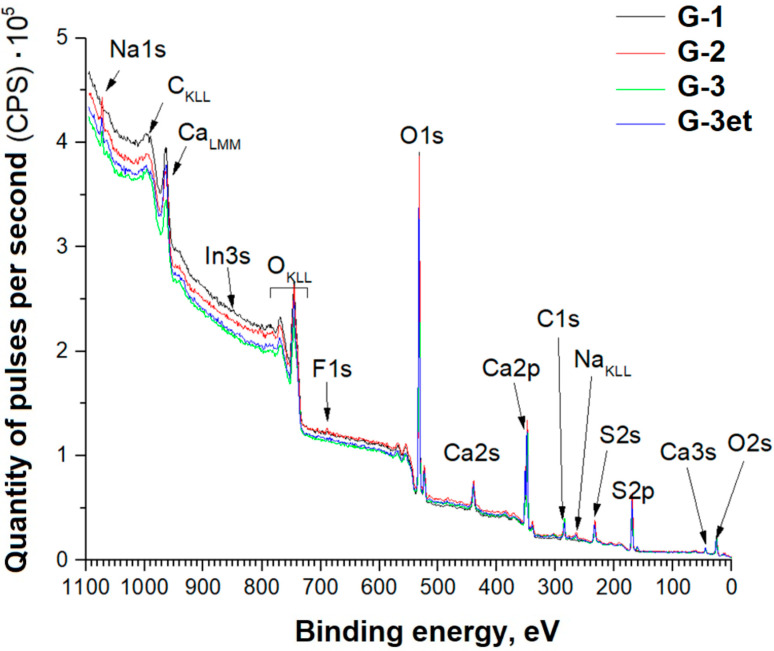
Panoramic spectra of calcium sulfate samples.

Panoramic XPS spectra of the sample series indicating the lines of the detected chemical elements are shown in Figure 9.

Based on the data obtained, the detected element concentrations in the gypsum samples’ (CaSO_4_·2H_2_O) surface layers were calculated (Table 2).

For easier perception, the samples are designated as follows:

G-1—original gypsum.

G-2—gypsum subjected to heat treatment at a temperature of 800 °C (temperature increase rate of 13 °C/min and isothermal holding for 60 min).

G-3—gypsum subjected to heat treatment in the presence of a reducing agent at the temperature of 800 °C (temperature increase rate of 13 °C/min and isothermal holding for 60 min).

G-3et—G-3 sample, additionally etched with argon ions with an energy of 1 keV (ion beam current of 10 mA) for 1 min.

**Figure 10:**
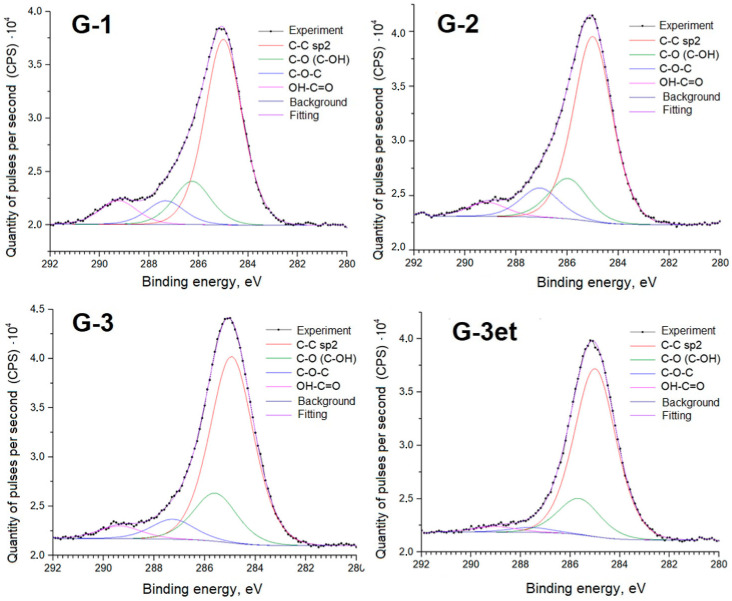
C1s spectra of calcium sulfate samples.

**Figure 11:**
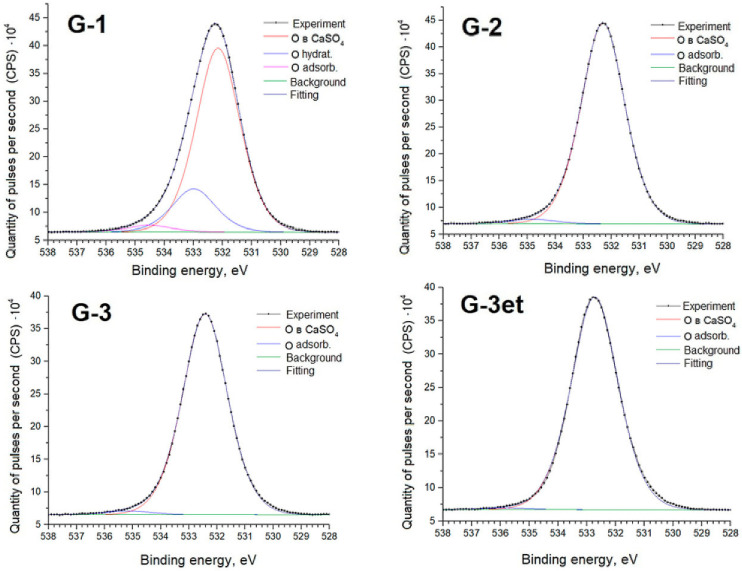
O1s spectra of calcium sulfate samples.

**Figure 12:**
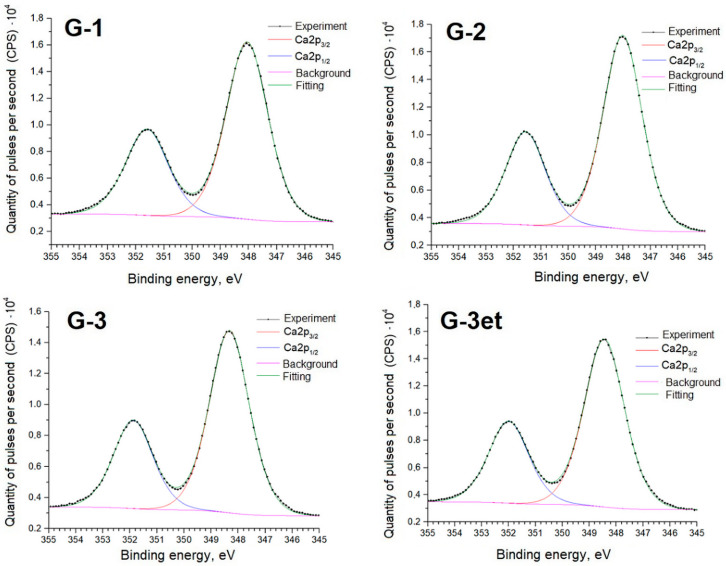
Ca2p spectra of calcium sulfate samples.

**Figure 13:**
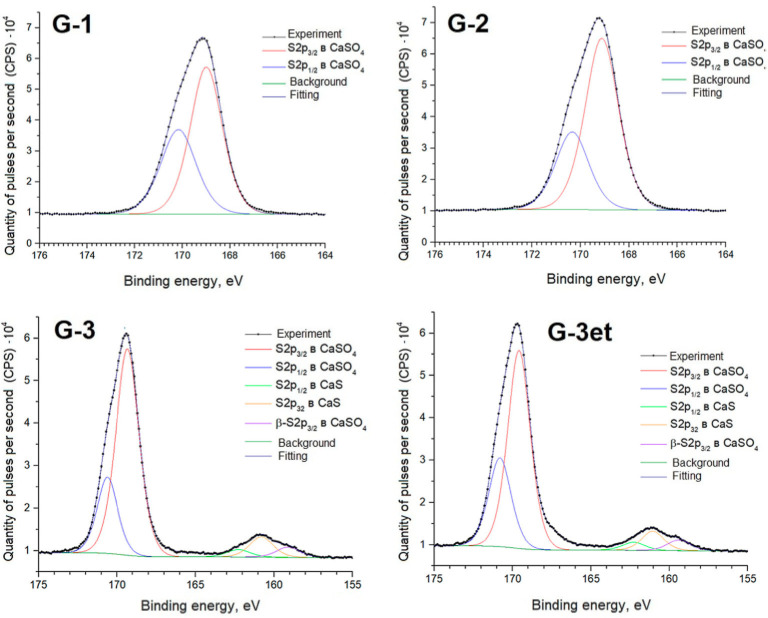
S2p spectra of calcium sulfate samples.

Below are detailed spectra of the samples and parameters of the spectra decomposition into components (Figure 10, Figure 11, Figure 12 and Figure 13) indicating the corresponding chemical bonds.

Similarly to the case of phosphogypsum, the C1s spectra of carbon adsorbed on the surface exhibit a set of lines standard for powder samples.

The O1s spectra exhibit lines (Eb = 534–535 eV) associated with the presence of adsorbed water. The G-1 sample spectrum exhibits a line (Eb = 533 eV) associated with water bound in the salt crystal hydrate.

The obtained spectra for calcium sulfate (both initial and heat-treated) are practically identical to the phosphogypsum samples’ spectra. The positions and intensity ratios of the Ca2p and S2p lines of samples G-1 and G-2 fully correspond to the reference values for calcium sulfate, CaSO_4_ [21]. In this compound, sulfur is in the oxidation state of +6. Sample G-3 additionally exhibits S2p lines related to calcium sulfide (CaS) (sulfur with an oxidation state of −2) [22]. The proportion of sulfate to sulfide is 90.8/9.2 before etching and 91.3/8.7 after argon ion etching.

### 2.3. Luminescent Ability

**Figure 14:**
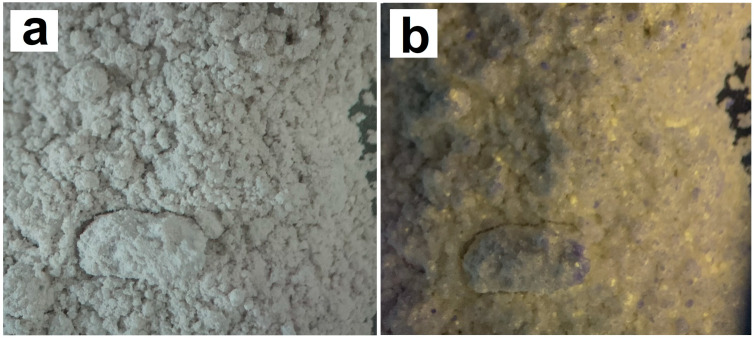
Photographs of phosphogypsum powders illuminated (**a**) with daylight and (**b**) with ultraviolet radiation of a 380 nm wavelength.

Heat-treated phosphogypsum in the presence of a reducing agent begins to exhibit the properties of an ultraviolet pigment: it emits in the yellow-orange range (Figure 14) when irradiated with light of a 380–395 nm wavelength.

It is noteworthy that we have experimentally established the fact that calcium, barium, and strontium sulfates exhibit similar properties: when treated in the presence of a reducing agent, the resulting composite materials of the MS/MSO_4_ (M = Ca, Sr, Ba) composition exhibit luminophore properties when exposed to UV radiation.

**Figure 15:**
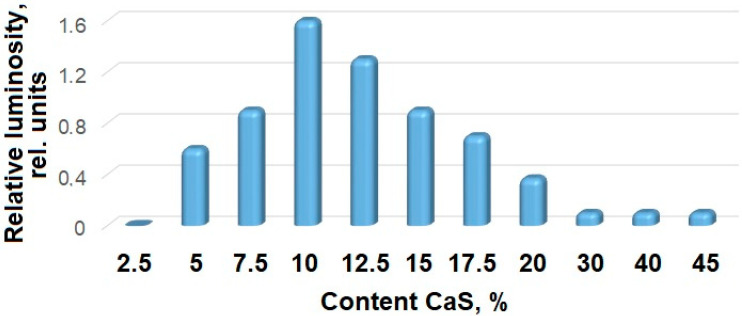
Samples’ relative luminosity dependence on the calcium sulfide content.

For phosphogypsum samples, the dependence of the sample luminosity on the content of the reduced phase (CaS) was studied, Figure 15. The experimental data indicate an extreme nature of the obtained materials’ luminosity dependence on the reduced phase content: with a small amount of reduced calcium sulfate, the luminosity of the samples increases with an increase in the CaS content, reaching maximum values at a calcium sulfide content of 7–15% (by weight, according to iodometric analysis). Then, with an increase in the amount of CaS, the luminosity decreases. This experimentally observed dependence can be associated with at least two factors: a change in the number of luminosity centers at the phase boundary; the luminosity increases with an increase in the number of radiative centers, passes the maximum value, and begins to decrease with an increase in defects leading to their recombination and a decrease in the proportion of the CaSO_4_ phase.

Some amounts of calcium sulfide formed during the reduction process will obviously remain part of the new phase grid and will be inseparable from it by common chemical methods. It is this sulfide/sulfate structure, which has a high defectiveness, that can be the source of luminescence centers. In this case, local areas with CaS can be considered as impurities in the crystal grid. It is widely known that luminescence properties can be associated with impurity defects [23,24,25]. Intrinsic structural defects of crystals are the components of luminescence centers and can be considered impurity defects [25,26].

Enhancement of luminescent activity [27] is observed upon charge carrier contact.

**Figure 16:**
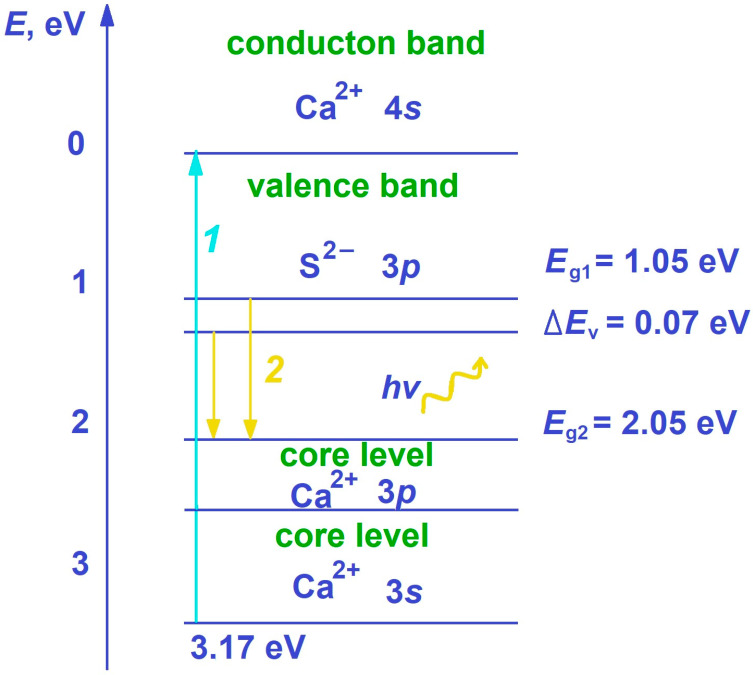
Possible mechanism for the radiative interzone transition formation in the optical range in the CaS/CaSO_4_ composite material.

Based on the obtained experimental data, the following scheme (Figure 16) can be proposed, explaining the possible mechanism of the radiative interzone optical transition formation in the samples. The intrinsic luminescence of several crystals is discussed in [26,28,29,30]. For some crystals of alkaline earth elements [31], a mechanism of crystal cross-luminescence in the ultraviolet region of the spectrum has been proposed. The essence of this phenomenon is associated with the formation of the conduction zone by the cation (Ca^2+^, 4*s*) atomic orbitals (vacant levels), and the valence zone is formed by the occupied p-levels of the anions (S^2−^, 3*p*) and the upper core levels of the cation (Ca^2+^, 3*p*, 3*s*). In this context, when the material is irradiated with photons with an energy of at least 3.17 eV, an electron and a vacancy in the 3*p* orbital are formed in the conduction zone (see Figure 3, transition 1 (blue arrow)). The peaks in the emission spectrum are the results of energy transitions between different states of the valence zone and the conduction zone of the material, which allows us to understand the processes that occur during the material excitation and light emission. Electrons from the valence zone fill the resulting vacancy, and a quantum of light is released—this is the manifestation of cross-luminescence (see Figure 3, transition 2 (yellow arrows)). These processes cause changes in the structure and properties of the material under the external radiation influence. The transitions occur from several states of the valence zone, so two distinct peaks with energies of 2.05 and 2.12 eV are observed in the luminescence spectrum.

A number of experiments were conducted to verify the proposed mechanism for luminescence center formation in reduced phosphogypsum. To confirm the presence of calcium sulfide in the PG-3 sample, a reaction was carried out between the suspension containing the sample and the copper (II) sulfate solution. After material drying, individual areas of black crystals were observed on the surface of the phosphogypsum. Presumably, transition reaction (7) of calcium sulfide (white) to copper (II) sulfide (black) occurred:

CaS + CuSO_4_ = CuS↓ + CaSO_4_ (7)

The reduced phosphogypsum sample was subjected to repeated heat treatment at a temperature of 1000 °C (the temperature increase rate was 13 °C/min with isothermal holding at the maximum temperature for 30 min). Upon completion, the samples were cooled with a furnace. As a result of the experiment, the material lost its luminescent properties. Presumably, this result is associated with the oxidation of calcium sulfide at elevated temperatures according to the reaction (8):

CaS + 2 O_2_ = CaSO_4_ (8)

## 3. Materials and Methods

### 3.1. Materials

Phosphogypsum (PG) according to the GOST R 58820-2020 (EuroChem Belorechenskiye LLC, Belorechensk, Russia) standard containing at least 92% of the main substance (CaSO_4_·2H_2_O) by dry weight and technical calcium sulfate CaSO_4_·2H_2_O (GOST 3210-77, EuroChem Belorechenskiye LLC, Belorechensk, Russia) were used for the synthesis of the materials. Sucrose (C_6_H_12_O_6_) was used as a reducing agent, in contrast to the previously conducted study [32].

### 3.2. Synthesis of Composite Materials

The experiment on reductive heat treatment of calcium sulfate was carried out according to the method described in detail in [32]. Briefly, phosphogypsum and calcium sulfate were pre-dried at 100 °C until constant weight was achieved. They were then carefully weighed on technical electronic scales with an accuracy of 0.01 g and then mixed with a reducing agent. After that, the mixture was placed in alundum crucibles and subjected to heat treatment at the temperature of 900 °C. Samples were cooled slowly and naturally until they reached room temperature.

Phosphogypsum (or calcium sulfate) and sucrose were used to prepare the samples, where the molar ratio of CaSO_4_/C was 1/2, following the previous studies [33]. It was found that this ratio of calcium sulfate and reducing agent is optimal.

### 3.3. Characteristics

The phase composition was investigated using an ARL X’TRA X-ray diffractometer (Thermo Fisher Scientific, Ecublens, Switzerland) with monochromatic Cu-Kα radiation by the point-by-point scanning method. The scanning step was 0.01°, and the data accumulation time at each point was 2 s. The range of measured values 2θ of the diffraction angle 2θ was from 5° to 90°.

Elemental analysis was performed using a Quanta 200 scanning electron microscope coupled with an EDAX Genesis XVS 30 X-ray microanalysis system (FEI Company, Hillsboro, OR, USA).

The calcium sulfide mass fraction in the reduced phosphogypsum sample was determined using the method described in [34]. For this purpose, a weighed sample of 500 mg of the reduced product was taken, placed in a vessel, and brought to 100 mL with deionized water. An aliquot of 10 mL was pipetted into a conical flask. A standard 0.1 N iodine solution of 10 mL was added and then 50 mL of deionized water was added. The mixture was acidified 1:1 with 2 mL of HCl and titrated with a standard 0.1 N sodium thiosulfate solution using a starch indicator until the solution became colorless.

The calcium sulfide mass fraction was determined using Formula (9):

(9) $${\omega (CaSO}_{4})=\frac{(V_{1}-V_{2})\cdot 36}{m_{m}}$$

where *V*_1_ is the volume of iodine solution added to the sample, mL; *V*_2_ is the volume of sodium thiosulfate used to titrate the remainder of unreacted iodine, mL; and *m_m_* is the mass of the reduced phosphogypsum weighed sample, mg.

Qualitative determination of calcium sulfide presence in the sample was carried out as follows: A 10 g reduced phosphogypsum sample was placed in a reaction vessel and filled with a copper (II) sulfate solution of a 0.1 mol/L concentration in a volume of 100 mL. The suspension was vigorously stirred for 5 min and then the precipitate was separated from the solution by filtration.

X-ray photoelectron spectroscopy (XPS) analysis was performed using a modernized SPECS X-ray electron spectrometer (SPECS GmbH—Surface Analysis and Computer Technology, Berlin, Germany) equipped with a gateway and a pretreatment chamber. Non-monochromatic X-ray radiation from a magnesium anode (hν = 1253.6 eV) was used. The Phoibos-150 (SPECS Surface Nano Analysis GmbH, Berlin, Germany) hemispherical deflector energy analyzer was used at constant pass energy mode of Epass = 15 eV. The binding energy scale (Eb) (C1s) calibration from the hydrocarbon layer is 285 eV. The analysis was performed under ultra-high vacuum conditions in the analytical chamber (at least 2 × 10^−9^ Torr; the composition of the residual medium was controlled using a gas mass spectrometer (SRS RGA-200, Sunnyvale, CA, USA)). No foreign impurity gases were detected in the vacuum system of the spectrometer. Some samples were etched with argon ions with an energy of 1 keV (ion beam current of 10 mA) for 1 min.

The element concentrations according to XPS data were determined considering the photoionization cross-sections of the corresponding electron levels. The sensitivity values were taken from the CasaXPS software (version 2.3.26) library [35]. The accuracy of concentration determination is ±3% of the measured value. The background was calculated using the Shirley method [36].

Samples for analysis were prepared by applying the powder onto an indium substrate by “rolling” it using a glass tube.

### 3.4. Study of Luminescent Properties

For each sample, the relative luminous flux emitted by the surface of a fixed-area sample was measured using an original setup described in [32] consisting of an ultraviolet (UV) radiation source, light filters, and a recording sensor. The sample under consideration and the reference sample, which was a yellow YAG:Ce luminophore, were placed in the setup and treated with radiation of a 380 nm wavelength; the voltage drop on the recording sensor was recorded, which occurred due to the sensor resistance change when the level of luminous flux on its surface changed. The relative luminous flux was obtained as the ratio of the voltage drop on the sensor when the sample under consideration was placed in the setup to the voltage drop on the sensor when the reference sample was placed in the setup.

## 4. Conclusions

It can be concluded that the luminescence of reduced phosphogypsum samples (as well as of calcium sulfate in the form of a chemical reagent) is associated with the CaS/CaSO_4_ composite material formation. During the reduction process, at the point of phase contact, the crystal grids are formed with a significant number of defects, which contribute to the convergence of some of the calcium cation and sulfide anion energy levels, facilitating the transition of electrons from the valence zone to the core zone and the formation of luminescence centers (cross-luminescence). When the composite material is destroyed (oxidation of calcium sulfide to sulfate) as well as when a large number of defects are formed, the luminescent properties disappear.

## Figures and Tables

**Table 1:** Element concentrations in the samples’ surface layers according to XPS data (at. %).

Sample	C	O	Ca	S	P	Si	Ru
PG-1	10.6	56.2	15.7	11.3	3.4	2.8	-
PG-2	8.9	56.6	15.9	15.1	1.7	1.7	0.1
PG-3	11.1	53.0	15.9	17.5	1.5	0.8	0.2
PG-3et	7.7	54.5	17.0	18.0	1.6	1.0	0.2

**Table 2:** Concentrations of elements in the gypsum samples’ surface layers according to XPS data (at. %).

Sample	C	O	Ca	S	P	Si	Ru
G-1	10.2	58.7	15.1	16.0	-	-	-
G-2	10.1	57.0	15.8	17.1	-	-	-
G-3	13.7	52.3	14.9	19.1	-	-	-
G-3et	10.0	54.2	15.8	20	-	-	-

## Data Availability

Data is contained within the article.
